# Gaining Surgical Access for Repositioning the Inferior Alveolar Neurovascular Bundle

**DOI:** 10.1155/2014/719243

**Published:** 2014-04-24

**Authors:** Saif Yousif Abdullah Al-Siweedi, P. Nambiar, P. Shanmuhasuntharam, W. C. Ngeow

**Affiliations:** ^1^Department of Diagnostic and Integrated Dental Practice, Faculty of Dentistry, University of Malaya, 50603 Kuala Lumpur, Malaysia; ^2^Department of Oro-Maxillofacial Surgical & Medical Sciences, Faculty of Dentistry, University of Malaya, 50603 Kuala Lumpur, Malaysia

## Abstract

This study is aimed at determining anatomical landmarks that can be used to gain access to the inferior alveolar neurovascular (IAN) bundle. Scanned CBCT (i-CAT machine) data of sixty patients and reconstructions performed using the SimPlant dental implant software were reviewed. Outcome variables were the linear distances of the mandibular canal to the inferior border and the buccal cortex of the mandible, measured immediately at the mental foramen (D1) and at 10, 20, 30, and 40 mm (D2–D5) distal to it. Predictor variables were age, ethnicity, and gender of subjects. Apicobasal assessment of the canal reveals that it is curving downward towards the inferior mandibular border until 20 mm (D3) distal to the mental foramen where it then curves upwards, making an elliptic-arc curve. The mandibular canal also forms a buccolingually oriented elliptic arc in relation to the buccal cortex. Variations due to age, ethnicity, and gender were evident and this study provides an accurate anatomic zone for gaining surgical access to the IAN bundle. The findings indicate that the buccal cortex-IAN distance was greatest at D3. Therefore, sites between D2 and D5 can be used as favorable landmarks to access the IAN bundle with the least complications to the patient.

## 1. Introduction

The inferior alveolar nerve (IAN) is the branch of the mandibular division of the trigeminal nerve that provides innervation to the mandible. Together with the inferior alveolar artery and vein, it enters the mandible via the mandibular foramen and runs an intraosseous course within the mandibular canal before dividing into two smaller branches, namely, the mental and the incisive nerves. It exits the mandible as the mental nerve via the mental foramen [1]. Having an in-depth knowledge of intraosseous position and course of the IAN is essential prior to commencing dental procedures in the mandible. This is because injury to the IAN has been reported to occur in restorative dentistry, endodontology, orthodontics, and, of course, oral and maxillofacial surgery [2, 3]. Oral and maxillofacial surgical procedures that are commonly associated with IAN injury range from minor oral surgical procedures, such as surgical removal of mandibular third molar [1], to more major surgeries, such as sagittal split osteotomy and mandibular jaw resection [4]. Lately, accidental encroachment of dental implants into the mandibular canal has been highlighted as another source of IAN injury [5].

The placement of endosseous dental implants in the posterior mandible to support fixed restorative prostheses is now a common treatment option for replacing missing molars. However, in cases where the alveolar bone has severely resorbed, sufficiently long fixtures cannot be placed without encroaching into the mandibular canal. Treatment options in such a case involve either the placement of short implant fixtures away from the mandibular canal, positioning of implants alongside the mandibular canal, onlay bone grafting to increase ridge height, alveolar distraction, or repositioning the IAN bundle to create adequate height for implant placement [6, 7]. The last approach, first described by Jensen and Nock [8], has been favored by some authors when dealing with severely resorbed mandible [7, 9].

Repositioning the IAN bundle, either by lateralization or fenestration, involves performing an osteotomy on the buccal cortex of the body of the mandible to create a window that enables the oral and maxillofacial surgeon to gain access to the IAN bundle. The IAN bundle will then be retracted more laterally to facilitate the insertion of long fixtures into the body of mandible [9]. This procedure is not without complication and, most often, short term (3–6 months) neurosensory dysfunction is the well-acknowledged risk that every patient has to take. Other unusual complications include pathological fracture to the body of mandible, presumably due to weakening of the mandible when excessive bone was removed [10]. IAN dysfunction happens because of the distension of the nerve during the surgical procedure or by compression/distension that follows [11]. Nevertheless, studies have shown that although this procedure resulted in a high percentage of minor IAN injuries (usually ischemia of the nerve), it provides a viable surgical procedure that allows implant placement in the resorbed posterior mandible with acceptable minor neurosensory alteration [11, 12].

Damage to the vital structures such as nerve and vessels and excessive removal of cortical bone may arise if clinicians merely use panoramic radiographs for presurgical planning. Hence, it is essential to have an in-depth knowledge of the location and the course of the mandibular canal as panoramic radiographs do not provide information on the horizontal (buccolingual) position of the IAN [13]. Recently, Pyun et al. [13] tried to predict the horizontal course of the mandibular canal by determining the location of the mental foramen on panoramic radiographs. The use of computed radiographic technology such as CT or CBCT is greatly advisable. In addition to preventing iatrogenic injury to the IAN, the information gathered will also avoid unnecessary bone removal that may further weaken a severely resorbed mandible.

It is the main aim of the present study to work out a safe zone for gaining access to the IAN bundle in a group of selected Asian populations. This is undertaken by firstly determining the presence and course of the mandibular canal by means of cone-beam computed tomography (CBCT), followed by determining the points of access for IAN repositioning. In essence, the specific objectives were

- to determine the presence (i.e., visualization) of the mandibular canal using CBCT,
- to measure the diameter of the mandibular canal at various predetermined points,
- to measure the apicobasal distance of the mandibular canal from the inferior border of the mandible at various predetermined points,
- to measure the buccolingual distance of the mandibular canal from the buccal cortex of the mandible at various predetermined points,
- to determine if the points of measurement can be used as landmarks to gain access to the IAN bundle,
- to determine if these anatomical landmarks are affected by the age, ethnicity, and/or gender of the subjects.

This study is undertaken using CBCT as it has proved to be a useful tool in detecting the mandibular canal [14] and negates the need to find cadaveric bodies of Malay samples as their Muslim religion advocated burial within 24 hours of demise.

## 2. Materials and Methods

### 2.1. Imaging

This study received the local Ethical Committee Board approval prior to commencement. Cone-beam computed tomography images were obtained with the i-CAT imaging system (Imaging Sciences International Inc., Hatfield, USA). All images were taken by the same technologist following a standardized protocol for patient positioning and exposure parameter setting (120 kVp, 3–7 mA, 20 sec) and image acquisition at 0.3 mm voxel size. Images were obtained from 100 consecutive patients referred to the Oral Radiology Division of the Faculty of Dentistry, University of Malaya. The following were the inclusion and exclusion criteria used for selecting suitable images.

*Inclusion Criteria.* They are as follows:

1. dentate adult patients between the ages 18 and 80 years,
2. healthy, medically compromised, or even previously radiated patients that did not involve the body of the mandible.

*Exclusion Criteria.* They are as follows:

1. patients with history of trauma or pathology to the mandible,
2. syndromic patients and patients with congenital disorders that affect the size of the jaw bones,
3. patients aged below 18 or above 80 years,
4. patients with history of surgical intervention to the body of the mandible, like orthognathic surgery or repair of a fracture,
5. patients of mixed racial origins,
6. patients with existing pathological disorder at mandible, such as cysts, tumours, osteomyelitis, and fibrous dysplasia,
7. the reformatted CBCT images, which appear distorted or blurred due to patients' movements.

### 2.2. Processing the Images

The visibility rating and dimensional measurements were performed by only one researcher (first author) who is trained in the interpretation of oral and maxillofacial images. Using cross-sectional images as described below, the visibility of the mandibular canal was categorized as “clearly visible, probably visible, or invisible” [15]. The DICOM data obtained were imported into SimPlant (SimPlant 3-D Pro version 13.1; Materialise Inc., Leuven, Belgium) and additional axial and/or sagittal images in the volume were analyzed if necessary. The SimPlant software allows viewing of axial, cross-sectional, panoramic, and 3D visualization of the jaw on the same screen.

All images were scored by the same observer, with randomly selected samples repeated again 2 weeks later to ensure reliability. Firstly, the mental foramen was identified and marked red. Then, the visible mandibular canal image was drawn onto the scan.

#### 2.2.1. Landmarks and Base Lines

Measurements were made for the direct linear distances between

**Figure 1:**
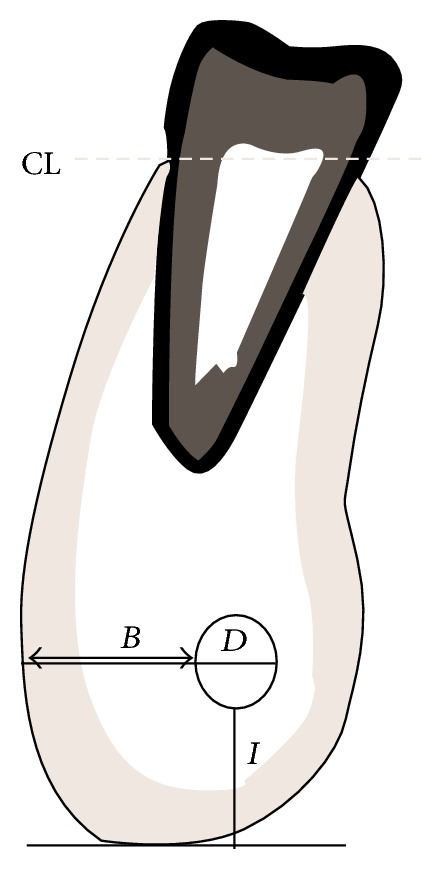
Cross-sectional sagittal view of a CBCT image showing the site for obtaining measurements. CL = crest of alveolar bone, *B* = distance between the mandibular canal and buccal cortex, *I* = distance between the mandibular canal and inferior body of mandible, and *D* = diameter of the mandibular canal.

- the buccal aspect of the mandibular canal and the outer buccal cortical margin of the mandible (Line *B*; Figure 1),
- the inferior aspect of the mandibular canal perpendicular to the inferior point of the lower border of the mandible (Line *I*; Figure 1).

**Figure 2:**
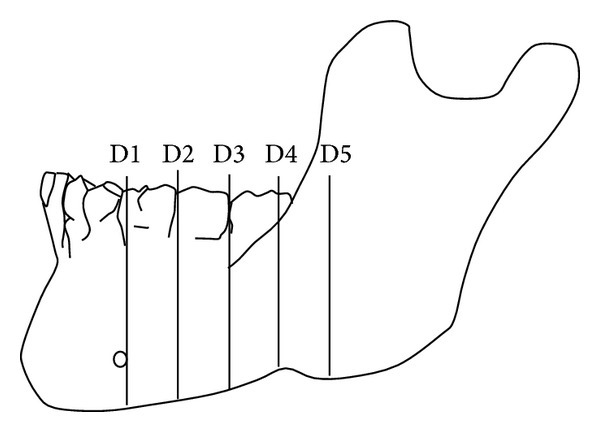
Illustration shows the locations of the measurements at every 10 mm interval starting from the distal aspect of mental foramen backwards (D1–D5). (D1) is the location of the mandibular canal at the distal aspect of mental foramen. (D2) is the location of the mandibular canal at 10 mm away from D1 distally. (D3) is the location of the mandibular canal at 10 mm away from D2 distally. (D4) is the location of the mandibular canal at 10 mm away from D3 distally. (D5) is the location of the mandibular canal at 10 mm away from D4 distally.

These measurements were done immediately at (D1) and at 10 (D2), 20 (D3), 30 (D4), and 40 (D5) mm distal to the mental foramen (Figure 2). In addition, the diameter of the mandibular canal was also measured at these points.

The widest diameter of the mandibular canal was measured on a horizontal plane (*D*), as shown in Figure 1.

Intrarater reliability was done by deriving Cronbach alpha coefficient for each of the 12 randomly selected records outcomes done at 2-week interval. Measurements error, that is, the difference between the corresponding measurements expressed in terms of *S*(*i*) values, was also calculated.

### 2.3. Statistical Analysis

All data were gathered, entered, and analyzed using SPSS 16.00 (SPSSFW, SPSS, Chicago, IL) software program. Descriptions of parameters were given as mean ± standard deviation (SD) and 95% confidence interval of mean. Independent *t*-test and correlation or analysis of variance (ANOVA) were used to determine the influence of site, age, gender, and ethnicity on the visibility of the mandibular canal and the course of the mandibular canal, respectively. Multiple regression analysis was then performed to determine which combination of predictors affects the results obtained. A 5% level of significance was chosen.

## 3. Results

The subjects for this study included images of 60 patients (30 males and 30 females) retrieved from the Division of Oral radiology, with ages of patients ranging from 20 to 60 years (mean age, 47 years). Forty other patients were excluded as they did not meet the inclusion criteria required.

The mandibular canal could be visualized clearly in all 60 CBCT images (120 sites). Independent *t*-tests showed that neither the side of image nor gender influences the visibility of the mandibular canal in the body of the mandible (*P* > 0.05). Furthermore, the age and ethnicity of the subjects also did not affect the visibility of the mandibular canal.

There was a good standardization and reproducibility of the base lines and measurements. The data analyzed for reliability of the measurements showed a Cronbach alpha coefficient of *r* = 0.91 and the value of the measurement errors, *S*(*i*), was 0.05 mm or less for all the compared data, which strongly suggested that the present method of obtaining measurements is considered reliable and accurate.

The side of mandible did not influence the obtained results. The mean distance of the mandibular canal from the lower border of the mandible was 10.09 ± 3.69 mm (95% CI = 9.79–10.39 mm). The apicobasal measurements obtained showed that the mandibular canal formed an elliptic-arc curve, similar to that described by Liu et al. [16] The mean inferior measurement was 9.37 ± 1.69 mm at D1, 8.24 ± 1.69 mm at D2, 7.96 ± 1.93 mm at D3, 9.66 ± 2.54 at D4, and 15.21 ± 4.18 mm at D5 (Table 1). The measurement taken at D3 was the closest to the inferior mandibular border as this point formed the lowest point of the elliptic-arc curve. This corresponds with the location around the second mandibular molar tooth.

The study showed that the gender of the subjects affects the distance of the mandibular canal to the inferior border of the mandible at D1, D2, and D3. The measurements were larger in males in all instances, that is, 10.10 ± 1.66, 8.96 ± 1.64, and 8.47 ± 1.93 mm in comparison to 8.64 ± 1.40, 7.92 ± 1.43, and 7.46 ± 1.82 mm for females.

**Figure 3:**
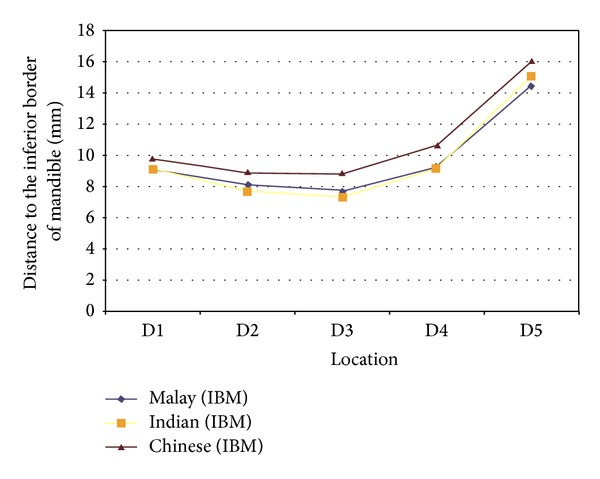
Distances of the mandibular canal to the inferior border of mandible immediately at (D1) and at 10 (D2), 20 (D3), 30 (D4), and 40 (D5) mm distal to the mental foramen of 3 different ethnic groups. An elliptic-arc curve pattern is observed when the results were put together.

Further analysis comparing the distances obtained for the three different ethnic groups shows all of them complying to the same elliptic-arc curve pattern (Figure 3). However, the mandibular canal of Chinese subjects was, in general, located significantly further away from the inferior border of the mandible when compared to the two other ethnic groups at the molar region (D3, ANOVA, *P* = 0.002 and D4, ANOVA, *P* = 0.002). However, further analysis using regression analysis suggests that race is not a predictor of the distance of the MC to the inferior border of mandible (*P* = 0.455). The main predictor that influences this distance is the age of the subject and it affects measurements at 4 locations. At D2 and D3, age and gender of the subjects affected the results obtained, while at D4 and D5, this was affected solely by the age of patients.

**Figure 4:**
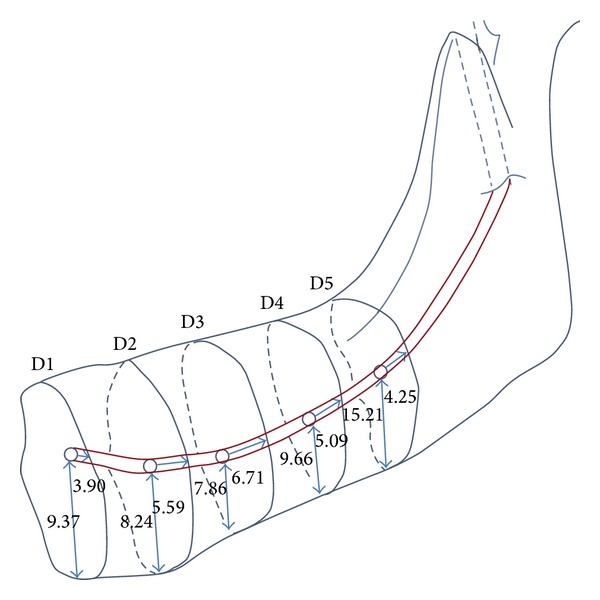
Distances of the mandibular canal to the buccal and inferior cortices of mandible immediately at (D1) and at 10 (D2), 20 (D3), 30 (D4), and 40 (D5) mm distal to the mental foramen of 3 different ethnic groups. A 3D elliptic-arc curve appearance is observed when both results were put together.

The mean distance from buccal cortex was 5.23 ± 1.71 mm (95% CI = 5.09–5.36 mm). The average buccolingual positions at D1, D2, D3, D4, and D5 were 3.90 ± 1.01 mm, 5.59 ± 1.20 mm, 6.71 ± 1.34 mm, 5.69 ± 1.63 mm, and 4.25 ± 1.60 mm, respectively. This also gives the mandibular canal an appearance of an elliptic-arc curve that spanned buccolingually. Taken together with the apicocoronal measurement described above, this gives the mandibular canal a 3D elliptic-arc curve appearance, as shown in Figure 4.

Unlike for the distance between the MC and the inferior border of mandible (IBM), the gender influence was noted only for one measurement on the buccal surface of the mandible; the measurement for males at 6.09 ± 1.70 mm was significantly higher than the females 5.28 ± 1.47 mm at 3 cm distal to the mental foramen (D4).

**Figure 5:**
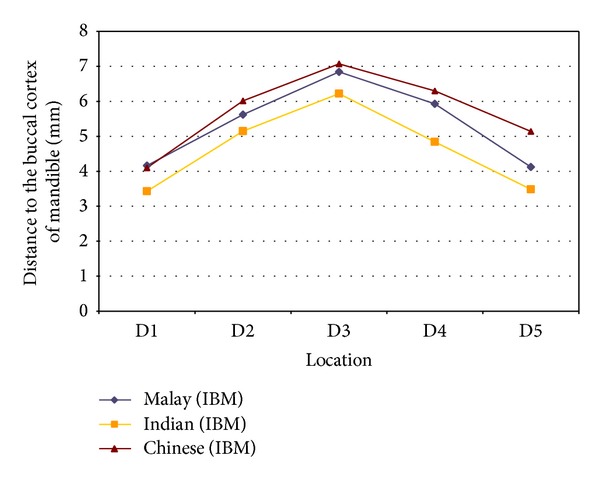
Distances of the mandibular canal to the buccal cortex of mandible immediately at (D1) and at 10 (D2), 20 (D3), 30 (D4), and 40 (D5) mm distal to the mental foramen of 3 different ethnic groups. A buccolingual elliptic-arc curve pattern is observed in relation to the buccal cortex.

However, the ethnicity of the subjects seems to exert a strong influence on the mean distances of the MC from the buccal cortex, as analyzed using analysis of variance (ANOVA, *P* < 0.001; Figure 5). Chinese subjects in general presented with measurements that were significantly furthest from buccal cortex of the mandible when compared to the other two ethnic groups. Their mandibular canal was located significantly further away than Indians at all 5 locations and also Malays at D5. In contrast, Indians presented with the shortest distance between the MC and buccal cortex (Figure 5).

Regression analysis shows that the ethnicity of the subjects affects all measurements from D1 to D5. In addition to it, the age of the subjects was influential at D1 and D3 and the age and gender of subjects were influential at D4.

Tables 2, 3, and 4 summarize various measurements obtained for all three ethnic groups of both genders. This information can become a handy guide for dental surgeon/oral surgeons needing to access the IAN in patients of these ethnic origins.

The overall mean size of the mandibular canal is 2.16 ± 0.44 mm (95% CI = 2.13–2.20 mm). As there was no ethnic difference in the size of the mandibular canal (ANOVA, *P* > 0.05), the data were pooled and are shown in Table 5. There is, however, gender difference, whereby the mean diameter of the mandibular canal of males was significantly larger than those of the females at all 5 points of measurement.

## 4. Discussion

The inferior alveolar neurovascular (IAN) bundle that is housed within the mandibular canal is an important anatomical structure within the body of mandible. It may need to be repositioned to provide adequate bone height for implant placement in cases of moderate to severely resorbed posterior mandible. However, this procedure increases the risk of neurosensory dysfunction and/or haemorrhage and thus raises the demand for proper presurgical assessment and planning beside the need to have an in-depth knowledge of the anatomy concerned. It was therefore the aim of this study to determine the position and course of the IAN bundle in Asian subjects as well as to determine possible surgical entry points because the latter information is missing. The findings will provide a guide for accessing the IAN that can avoid causing iatrogenic nerve damage and, at the same time, provide the ability to gauge the amount of bone needed to be removed without weakening the mandible.

This study employs CBCT images instead of using cadaveric specimens as the visibility of the mandibular canal and the marginal crest as well as the observer agreement of the location of these structures has been reported to be high [14]. Studies have shown that measurements obtained from CBCT images are also comparable to direct cadaveric measurements [17]. In addition, Maloney et al. [18] have shown that results obtained using SimPlant dental software were accurate and not different from direct cadaveric measurement or measurement using the original i-CAT CBCT software. This approach will negate the need for Malay cadavers as they are impossible to obtain due to their Muslim religion affiliation that requires deceased persons to be buried within 24 hours. Malay, Indian, and Chinese subjects were included in this study as they represent a majority of the Asian population (Malay: Indonesia and Malaysia, 250 million; Indian: India, 1.2 billion; Chinese: China, 1.33 billion).

In this study, all measurements were made on buccal and inferior surfaces of the basal bone where the mandibular canal is located. This area undergoes less resorption as compared to alveolar bone. A study has shown that the distance of the mandibular canal to the external lingual and buccal cortical plates remained remarkably constant with increasing atrophy [19]. Thus, the authors believe that the results obtained were consistent, regardless of whether the patients were dentate or edentulous. However, further studies with bigger sample sizes are needed to confirm the suggestion that the lack of teeth in the part of the jaw does not influence the results obtained. This is because Kilic et al. [20] reported different measurements in subjects who were dentate, partially dentate, and edentulous. The inferior border of the mandible was used as a base for measurement as several studies have shown that it is a valid reference point to determine the course of the mandibular canal [15, 21, 22].

Analysis comparing the left and right side of the mandible shows that the results were not different, confirming previous findings that suggested the symmetrical appearance of anatomical structures of the mandible [23]. All mandibular canals follow an elliptic-arc curve as described by Liu et al. [16] or the catenary-like configuration described by Worthington [24] and Ozturk et al. [25]. The mean distance of the mandibular canal from the inferior border of mandible, as reported here (10.09 ± 3.69 mm), is slightly shorter than the mean of 10.52 mm reported by Kilic et al. [20] but is similar to the distance of 10 mm described by Gowgiel [22], Rajchel et al. [21], and Ozturk et al. [25]. Yu and Wong [26] have reported a shorter mean inferior distance of 7.6 mm at the second molar region in Chinese, which corresponded with site D3 in our subjects. At 7.96 ± 1.93 mm, the measurement at D3 is also close to the distance of 7.56 ± 1.62 mm reported by Liu et al. [16] for the same parameter at the apical area of the mandibular first molar. The finding suggests that the shortest distance between the mandibular canal and the inferior border of the mandible in our subjects was located more posteriorly than that reported in the literatures but is in agreement with a recent report on Singaporean Chinese [27]. In comparison, Wang et al. [28] and Liu et al. [16] stated that the distance of the mandibular canal to the inferior border of the mandible was the shortest in the area corresponding to the apical area of the second premolar and mandibular first molar, respectively. The findings of this study indicate that there are variable distances between the mandibular canal and the lower border of mandible and this can be influenced by the gender and age of the subjects. This is in contrast to the findings by Ozturk et al. [25] who reported that apicobasal measurements did not vary according to race, gender, or age.

Differences in inferior measurements at all 5 points of interest resulted in the mandibular canal following an elliptic-arc curve appearance as described by Liu et al. [16]. This is in agreement with the pattern reported by other researchers [21, 23]. In contrast, Kilic et al. [20] found no differences for this distance among different levels of measurements.

The main reference point of this study was the mental foramen and it could be used as a landmark to identify the beginning of the IAN bundle in patients with missing posterior teeth. At 9.37 mm from the inferior border of the mandible, this distance is within the range of those reported in the literature, that is, 9.2 mm by Hwang et al. [29], 11.51 mm by Kilic et al. [20], and 12.0 mm by Neiva et al. [30]. However, Kilic et al. [20] reported that this distance varied among the dentate, partially dentulous, and edentulous groups, being 8.90, 10.30, and 12.33 mm, respectively. Further studies need to be done to ascertain whether such a similar situation is seen in these 3 ethnic Asian groups.

The average distance between the mandibular canal and the buccal cortex reported here (5.23 ± 1.71 mm) is higher than those reported by other authors: 4.9 mm by Levine et al. [1], 5.0 mm by Gowgiel [22], and 4.58 mm by Kilic et al. [20]. However, the average distances reported by Kilic et al. [20] in a combination sample were (a) the dentate group (mean 5.80 mm); (b) the partially dentulous group (7.85 mm); and (c) the edentulous group (5.43 mm). So, if comparison is done solely for those with dentition, the distance reported by Kilic et al. [20] was in fact larger than those reported in this study. In agreement with other authors, the distance between the mandibular canal and buccal cortex varies from the second premolar to the second molar region. Comparison shows that measurements obtained in Koreans and Taiwan Chinese subjects were larger. Hwang et al. [29] reported a higher measurement for the distances at the second premolar (4.3 mm), first molar (6.8 mm), and second molar (8.3 mm) than what was reported here (D1/PM1: 3.90 mm; D2/M1: 5.59 mm; and D3/M2: 6.71 mm). Yu and Wong [26] also reported a bigger distance of 7.2 mm for the last parameter in Taiwan Chinese. However, our distance at the second molar is similar to the 6.79 mm reported in Singaporean Chinese, which coincidently is also the furthest point from the buccal cortex of their subjects [27]. In comparison, Ozturk et al. [25] recently reported shorter distances at the first and second molar in skulls of different races. These differences may have resulted from the variations in age and ethnicity of the subjects included. Levine et al. [1] reported that age and ethnicity were statistically associated with position of the mandibular canal in relation to the buccal cortex. They found that older patients and white patients, on average, showed less distance. This current study found the same association.

The overall maximum mean size of the mandibular canal of 2.16 ± 0.44 mm is within the range of 2.0 to 2.4 mm reported by Rajchel et al. [21] in a cadaveric study of Asian subjects. However, other authors reported higher figures of 2.52 mm [20] to as much as 3.4 mm wide [31] depending on the location and method of measurement (vertical versus horizontal measurement). In fact, Hur et al. [32] even reported a width of 4.1 mm at the retromolar region.

The 3D reconstruction of the mandibular canal against the inferior and buccal borders of the mandible (Figure 4) suggests that the mandibular canal present as an elliptic-arc curve is not only at one plan (apicobasal), but also at the buccolingual plan. Liu et al. [16] reported that the elliptic-arc curve pattern is the most common pattern observed in panoramic radiographs, but their study was not able to determine the buccolingual orientation of the IAN bundle. Kim et al. [15] reported that the buccolingual orientation of the mandibular canal followed either of these 3 different patterns.

 Type 1 (70%): the canal follows the lingual cortical plate at the mandibular ramus and body.
 Type 2 (15%): the canal follows the middle of the ramus behind the second molar and the lingual plate passing through the second and first molars.
 Type 3 (15%): the canal follows the middle or the lingual one-third of the mandible from the ramus to the body.

**Figure 6:**
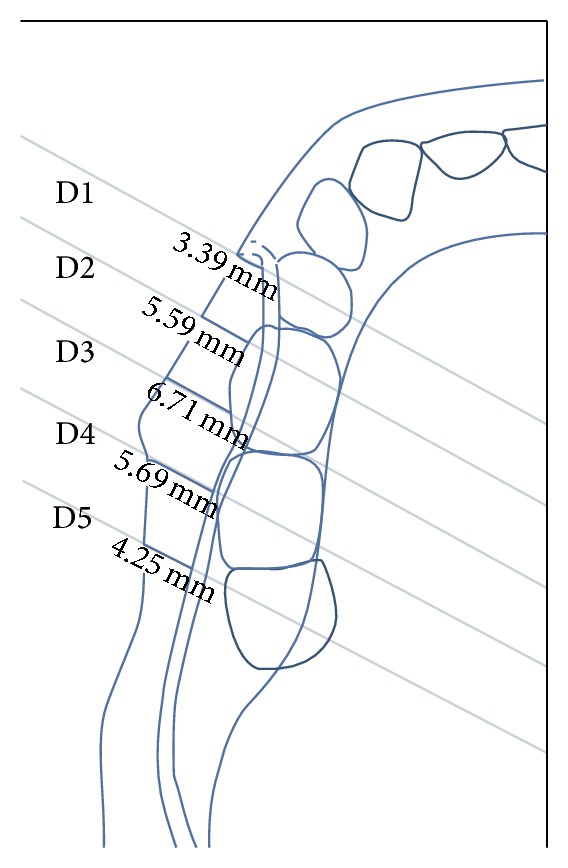
Superimposition of the findings of this study over a curved body of mandible displays differences in distance between various points of the MC to the buccal cortex.

Hwang et al. [29] found the mandibular canal to be nearer to the lingual side in the posterior two-third of the mandible, but nearer to the buccal side in the anterior one-third. Such a finding may possibly be observed for the mental region and anterior part of the body of the mandible in this study. However, caution must be expressed as the buccal cortex of the mandible is not a flat surface; hence, in reality, the longer distance between the MC and the buccal cortex is a result of an outward curvature over the body of the mandible, as shown in Figure 6.

Two inferior alveolar nerve repositioning techniques have been described, namely, lateralization and fenestration [7]. In lateralization, drilling is done around the mental foramen to obtain a ring of external cortical bone. An extension of about 5 mm is made anteriorly to the mental foramen to avoid damaging the anterior loop and another posterior window is performed along the intrabony trajectory of the nerve. The incisive nerve that is located about 5 mm from the mental foramen needs to be sectioned in order to secure complete mobilization of the alveolar nerve [6]. In the case of fenestration, sectioning of the incisive nerve is not performed. This approach only involves the preparation of a cortical bone window located posterior to the mental foramen. While both techniques described the approach that can be used to reposition the IAN bundle, recent journal reports do not describe the anatomical landmarks that can be used for guidance.

So, in order to perform IAN repositioning in Asians, it is suggested that the surgeons first locate the mental foramen based on available radiographs. The most common location for the mental foramen in some Asian ethnic groups such as Malay, Indians, and Chinese has been reported to be at apical to the second premolar [33–35]. An osteotomy should be done with a margin of about 2 mm around the mental foramen to free the nerve. This can be done by using a Lindemann or stainless steel surgical bur with a diameter of 1.5 to 2 mm [1] to a depth that is similar to the entire diameter of the bur. There should not be any worry of causing iatrogenic injury to the IAN bundle beneath as the mean distance from the buccal margin to the canal is almost 4 mm. Adopting results of this study, this inferior cut should be done 7 mm from the inferior border of the mandible.

Following this, position D3, which is 20 mm distal to the mental foramen, is identified using a metal ruler/caliper. This is the site where the mandibular canal presented with the shortest distance (7.96 ± 1.93 mm) to the lower border of mandible and concurs to the region of the second molar. A round surgical stainless steel bur can be used to make this second osteotomy mark, which should be about 6 mm from the inferior border of the mandible. This distance will avoid cutting into the lower cortical plate that has been reported to range between 3.2 and 3.5 mm in thickness in Asian subjects [36]. Later on, position D2 is located and an osteotomy mark is made about 6 mm from the lower border of the mandible. Subsequent markings at position D4 can be made also at 9 mm from the lower border of mandible, if necessary. Chinese subjects seemed to have a mandibular canal that is located further from the inferior border of mandible than Malay or Indian subjects, so this fact needs to be given due consideration during the making of osteotomy marks. This can be done by confirming radiographic findings prior to IAN bundle repositioning.

The depth of drilling for making osteotomy marks should be between 2.5 and 3.0 mm as studies have shown that these are the thicknesses of buccal plates of Asians at the molar regions [36]. One is unlikely to injure the IAN at these depths as the current results show that the mandibular canal is located between 3.90 and 6.71 mm from the buccal cortex.

Considering the fact that the maximum mean diameter of the mandibular canal is 2.25 mm, corresponding osteotomy marks on the superior margin should be made 6 mm superior to the inferior osteotomy marks. These osteotomy marks can then be joined using an ultrasound bone surgical device as it has proven to spare injuring nerves, in case variations happen with the location of the IAN bundle [37]. At this size, this osteotomy window should be small enough to not weaken the mandible, but adequate to facilitate bone repair. This also ensures that there is adequate bone tissue above the canal so that the primary stability needed for implant insertion is not compromised [38]. If lateralization of the IAN bundle is necessary, further osteotomy is made into the buccal cortex at 5 mm mesial to the mental foramen, following the recommendation by Morrison et al. [6].

This study found that the IAN follows an elliptic-curve arc course in relation to the curved body of the mandible, so one needs to ensure that more cancellous bone is removed at D3 (i.e., 20 mm or around the region of the mandibular second molar) in order to reach the IAN. The findings of this study suggested that there are some ethnic features that influence the horizontal location of the mandibular canal; hence, they are of importance in clinical practice. For example, the IAN of Chinese subjects is located further from the buccal cortex than Indian subjects. Therefore, more bone needs to be removed for Chinese subjects than Indian or Malay subjects. Further studies with larger sample size are needed to verify these suggestions.

## 5. Conclusion

The mandibular canal was visible in all (100%) CBCTs, that is, 120 sites. The ease of detection of the mandibular canal using CBCT and SimPlant dental software indicates the potentially high preoperative value of CBCT scan for the purpose of preoperative planning. Apicobasal assessment of the canal reveals that it was curving downward towards the inferior mandibular border until 20 mm distal to the mental foramen (D3) and then reverts upwards, making an elliptic-arc curve. The mandibular canal also forms an elliptic arc in relation to the curved body of mandible, with the furthest buccal point located at D3. While acknowledging that there is human variability, this study provides an accurate anatomic location of the mandibular canal, which in return helps to determine a safe zone to access the IAN bundle. This hopefully will become a useful guide in centers where CBCT is not available. When such a facility is available, it is recommended that clinicians make use of it to overcome the shortfalls observed in conventional radiography.

## Figures and Tables

**Table 1:** Mean measurement with standard deviation and the corresponding 95% confidence interval for the distances of the mandibular canal to the inferior border and buccal cortex of the mandible.

Position	Mean ± SD; 95% confidence interval of distance from the mandibular canal to two cortical borders of mandible, namely,	
	Inferior	Buccal
D1	9.37 ± 1.69 (9.07–9.68) mm	3.90 ± 1.01 (3.71–4.08) mm
D2	8.24 ± 1.69 (7.93–8.55) mm	5.59 ± 1.20 (5.38–5.81) mm
D3	7.96 ± 1.93 (7.61–8.31) mm	6.71 ± 1.34 (6.47–6.95) mm
D4	9.66 ± 2.54 (9.20–10.12) mm	5.69 ± 1.63 (5.39–5.98) mm
D5	15.21 ± 4.18 (14.46–15.97) mm	4.25 ± 1.60 (3.96–4.54) mm
Overall	10.09 ± 3.69 (9.79–10.39) mm	5.23 ± 1.71 (5.09–5.36) mm

**Table 2:** Mean measurement with standard deviation and corresponding 95% confidence interval for the distances of the mandibular canal to the inferior border (IBM) and buccal cortex in Malay subjects.

Site	Gender		Overall
	Male	Female	
D1			
IBM^*α*^	10.30 ± 1.36 mm (9.67–10.94 mm)	7.95 ± 1.50 mm (7.26–8.66 mm)	9.13 ± 1.84 mm (8.54–9.72 mm)
Buccal	4.17 ± 0.99 mm (3.70–4.63 mm)	4.15 ± 1.06 mm (3.65–4.65 mm)	4.16 ± 1.02 mm (3.83–4.48 mm)
D2			
IBM^*β*^	9.32 ± 1.36 mm (8.68–9.95 mm)	6.92 ± 1.14 mm (6.38–7.45 mm)	8.12 ± 1.74 mm (7.56–8.67 mm)
Buccal	5.64 ± 1.07 mm (5.14–6.14 mm)	5.60 ± 1.13 mm (5.07–6.14 mm)	5.62 ± 1.09 mm (5.28–5.97 mm)
D3			
IBM^*γ*^	8.99 ± 1.25 mm (8.40–9.57 mm)	6.43 ± 1.37 mm (5.79–7.07 mm)	7.71 ± 1.83 mm (7.12–8.29 mm)
Buccal*	7.24 ± 0.92 mm (6.81–7.67 mm)	6.44 ± 1.39 mm (5.79–7.09 mm)	6.84 ± 1.23 mm (6.45–7.24 mm)
D4			
IBM^*δ*^	10.46 ± 2.01 mm (9.52–11.40 mm)	8.03 ± 2.94 mm (6.66–9.41 mm)	9.25 ± 2.78 mm (8.36–10.13 mm)
Buccal**	6.65 ± 1.21 mm (6.08–7.21 mm)	5.20 ± 1.65 mm (4.43–5.98 mm)	5.93 ± 1.61 mm (5.41–6.44 mm)
D5			
IBM	15.03 ± 4.14 mm (13.09–16.97 mm)	13.96 ± 4.12 mm (12.03–15.89 mm)	14.49 ± 4.11 mm (13.18–15.81 mm)
Buccal^§^	4.44 ± 0.93 mm (4.01–4.88 mm)	3.80 ± 0.86 mm (3.40–4.21 mm)	4.12 ± 0.94 mm (3.82–4.43 mm)

^*α*^Independent *t*-test, *P* < 0.001.

^*β*^Independent *t*-test, *P* < 0.001.

^*γ*^Independent *t*-test, *P* < 0.001.

^*δ*^Independent *t*-test, *P* = 0.005.

*Independent *t*-test, *P* = 0.039.

**Independent *t*-test, *P* = 0.003.

^§^Independent *t*-test, *P* = 0.031.

**Table 3:** Mean measurement with standard deviation and corresponding 95% confidence interval for the distances of the mandibular canal to the inferior border (IBM) and buccal cortex in Indian subjects.

Site	Gender		Overall
	Male	Female	
D1			
IBM*	9.81 ± 2.03 mm (8.86–10.76 mm)	8.51 ± 1.26 mm (7.92–9.10 mm)	9.16 ± 1.79 mm (8.58–9.73 mm)
Buccal	3.65 ± 0.82 mm (3.24–4.05 mm)	3.21 ± 0.88 mm (2.80–3.62 mm)	3.43 ± 0.89 mm (3.15–3.71 mm)
D2			
IBM	8.17 ± 1.75 mm (7.35–9.00 mm)	7.26 ± 1.41 mm (6.60–7.92 mm)	7.72 ± 1.64 mm (7.19–8.24 mm)
Buccal	5.31 ± 1.37 mm (4.67–5.95 mm)	4.99 ± 1.11 mm (4.47–5.51 mm)	5.15 ± 1.24 mm (4.75–5.55 mm)
D3			
IBM	7.34 ± 2.29 mm (6.27–8.41 mm)	7.38 ± 1.81 mm (6.54–8.23 mm)	7.36 ± 2.04 mm (6.71–8.01 mm)
Buccal	6.34 ± 1.67 mm (5.56–7.12 mm)	6.10 ± 0.76 mm (5.74–6.46 mm)	6.22 ± 1.29 mm (5.81–6.63 mm)
D4			
IBM	8.62 ± 2.95 mm (7.24–10.00 mm)	9.65 ± 2.03 mm (8.70–10.60 mm)	9.14 ± 2.55 mm (8.32–9.95 mm)
Buccal	5.26 ± 1.66 mm (4.48–6.03 mm)	4.42 ± 0.89 mm (4.00–4.84 mm)	4.84 ± 1.38 mm (4.40–5.28 mm)
D5			
IBM	14.25 ± 5.32 mm (11.76–16.75 mm)	15.95 ± 4.02 mm (14.07–17.83 mm)	15.10 ± 4.74 mm (13.59–16.62 mm)
Buccal	3.36 ± 1.69 mm (2.57–4.14 mm)	3.60 ± 1.51 mm (2.89–4.31 mm)	3.48 ± 1.59 mm (2.97–3.98 mm)

*Independent *t*-test, *P* = 0.021.

**Table 4:** Mean measurement with standard deviation and corresponding 95% confidence interval for the distances of the mandibular canal to the inferior border (IBM) and buccal cortex in Chinese subjects.

Site	Gender		Overall
	Male	Female	
D1			
IBM	10.18 ± 1.56 mm (9.45–10.91 mm)	9.47 ± 1.02 mm (8.99–9.95 mm)	9.83 ± 1.35 mm (9.40–10.26 mm)
Buccal	4.35 ± 1.11 mm (3.83–4.87 mm)	3.85 ± 0.74 mm (3.50–4.19 mm)	4.10 ± 0.97 mm (3.79–4.41 mm)
D2			
IBM*	9.40 ± 1.54 mm (8.68–10.13 mm)	8.40 ± 1.34 mm (7.77–9.03 mm)	8.90 ± 1.52 mm (8.42–9.39 mm)
Buccal	6.06 ± 1.13 mm (5.54–6.59 mm)	5.96 ± 1.17 mm (5.41–6.50 mm)	6.01 ± 1.13 mm (5.65–6.37 mm)
D3			
IBM	9.08 ± 1.67 mm (8.30–9.86 mm)	8.56 ± 1.64 mm (7.79–9.33 mm)	8.82 ± 1.65 mm (8.29–9.35 mm)
Buccal	7.09 ± 1.53 mm (6.37–7.80 mm)	7.06 ± 1.26 mm (6.47–7.64 mm)	7.07 ± 1.38 mm (6.63–7.51 mm)
D4			
IBM	10.50 ± 1.94 mm (9.59–11.41 mm)	10.69 ± 2.20 mm (9.66–11.72 mm)	10.60 ± 2.05 mm (9.94–11.25 mm)
Buccal	6.37 ± 1.90 mm (5.48–7.26 mm)	6.23 ± 1.20 (5.67–6.80)	6.30 ± 1.57 mm (5.80–6.80 mm)
D5			
IBM	15.81 ± 3.10 mm (14.35–17.26 mm)	16.30 ± 4.04 mm (14.41–18.20 mm)	16.06 ± 3.57 mm (14.92–17.20 mm)
Buccal**	4.44 ± 1.51 mm (3.73–5.15 mm)	5.85 ± 1.64 mm (5.08–6.61 mm)	5.14 ± 1.71 mm (4.60–5.69 mm)

*Independent *t*-test, *P* = 0.035.

**Independent *t*-test, *P* = 0.007.

**Table 5:** Mean measurement with standard deviation and 95% confidence interval for the diameter of the mandibular canal.

Site	Gender		Overall
	Male	Female	
D1*	2.38 ± 0.39 mm (2.28–2.48 mm)	2.14 ± 0.53 mm (2.00–2.28 mm)	2.26 ± 0.48 mm (2.17–2.35 mm)
D2**	2.20 ± 0.44 mm (2.09 ± 2.32 mm)	1.82 ± 0.30 mm (1.74–1.90 mm)	2.01 ± 0.42 mm (1.93–2.09 mm)
D3^§^	2.25 ± 0.34 mm (2.16–2.33 mm)	1.96 ± 0.35 mm (1.87–2.05 mm)	2.10 ± 0.37 mm (2.04–2.17 mm)
D4^¶^	2.36 ± 0.41 mm (2.26–2.47 mm)	2.00 ± 0.35 mm (1.91–2.09 mm)	2.18 ± 0.42 mm (2.11–2.26 mm)
D5^*γ*^	2.34 ± 0.50 mm (2.22–2.47 mm)	2.17 ± 0.33 mm (2.08–2.25 mm)	2.25 ± 0.43 mm (2.18–2.33 mm)

*Independent *t*-test, *P* = 0.005.

**Independent *t*-test, *P* < 0.001.

^§^Independent *t*-test, *P* < 0.001.

^¶^Independent *t*-test, *P* < 0.001.

^*γ*^ Independent *t*-test, *P* = 0.024.
